# One Species Hibernates Shorter, the Other Longer: Rapid but Opposing Responses to Warming Climate in Two Sympatric Bat Species

**DOI:** 10.1111/gcb.70531

**Published:** 2025-10-02

**Authors:** Gabriella Krivek, Frauke Meier, Leo Grosche, Gerald Kerth, Jaap van Schaik

**Affiliations:** ^1^ Zoological Institute and Museum, Applied Zoology and Nature Conservation University of Greifswald Greifswald Germany; ^2^ Niedersächsischer Landesbetrieb für Wasserwirtschaft Küsten‐ und Naturschutz Hannover Germany

**Keywords:** climate change, hibernation phenology, *Myotis daubentonii*, *Myotis nattereri*

## Abstract

The timing of hibernation represents a key seasonal transition in the annual cycle of hibernators, directly impacting their survival and reproductive success. Ongoing climate change affects many of the factors that influence hibernation phenology, such as weather patterns, food availability and the timing of mating and reproduction. The extent to which individuals should adjust their hibernation phenology in response is likely to vary between species, sexes and age classes. Consequently, long‐term, individualised datasets are essential for capturing individual responses to climate change and understanding the underlying mechanisms. However, such datasets remain exceedingly rare for wild hibernators. Using 13 years of individual‐level RFID‐logging data from over 4000 marked bats, we discovered strikingly different shifts in hibernation phenology in two sympatric species. While *Myotis nattereri* shortened its hibernation period with warming winters, *Myotis daubentonii* surprisingly prolonged its hibernation duration. These opposing trends were primarily driven by shifts in hibernation entry in autumn. Within both species, phenological shifts varied by sex and age, with adult males exhibiting the most pronounced changes. In *M. daubentonii* , adult males extended the duration of hibernation by 1.8 days/year, equating to almost a full month over the study period. In *M. nattereri* , adult males reduced the duration of hibernation by 2.3 days/year, resulting in a one‐third decrease in total hibernation duration. These remarkable shifts were strongly correlated with autumn temperatures: for each 1°C rise, hibernation entry in *M. daubentonii* advanced by 9.3 days, whereas in *M. nattereri* it was delayed by 6.2 days. Our results highlight that hibernation phenology can shift rapidly and in unforeseen, species‐specific ways in response to climate change. As mismatches between hibernation timing and prey availability can impact survival and reproduction, these phenological shifts could significantly affect individual fitness and population viability.

## Graphical abstract

Hibernation timing is a crucial seasonal transition that directly influences the survival and reproduction of hibernating mammals. Climate change alters key drivers of hibernation phenology, including temperature, food availability, and reproductive timing, but individual responses are expected to vary across species, sexes and age classes. Using 13 years of individual‐level RFID data from more than 4000 bats, we documented strikingly different responses in two sympatric species: *Myotis nattereri* shortened its hibernation, while *M. daubentonii* surprisingly prolonged its hibernation duration. These changes were primarily driven by shifts in autumn entry and were most pronounced in adult males, amounting to nearly a month of change over the study period. The phenological shifts were strongly linked to autumn temperatures, with *M. daubentonii* entering earlier and *M. nattereri* later in warmer years. Our findings demonstrate that hibernation phenology can shift rapidly and in unforeseen species‐specific ways, with potential consequences for individual fitness and long‐term population viability.

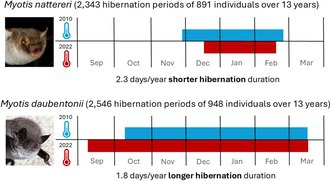

## 1 Introduction

Climate change is altering the timing of seasonal biological events, such as migration, hibernation and reproduction, across a wide range of taxa and ecosystems (Parmesan and Yohe 2003; Thackeray et al. 2016). In temperate and cold environments, hibernation is a key phenological trait for many mammals, enabling them to survive adverse winter conditions (Geiser 2021). The timing of hibernation (i.e., entry into and emergence from a hibernaculum) affects both survival probability during winter (Cordes et al. 2020; Ozgul et al. 2010) and reproductive success in the following summer (Lane et al. 2012). Climate change may alter the optimal timing of hibernation, either directly through seasonal changes in temperature or precipitation (Inouye et al. 2000) or indirectly through shifts in the phenology of predators or prey (Boyles et al. 2024). If hibernators are unable to keep pace with these changes, mismatches between hibernation timing and food availability or reproduction could have severe demographic consequences, particularly for species with slow life histories such as most hibernating mammals (Turbill et al. 2011).

Hibernation phenology is shaped by multiple ecological and physiological factors. At its core, hibernation is an adaptation to seasonal periods of energetic stress, when energy demands are greater than energy availability in the environment (Vuarin and Henry 2014). Therefore, in cases where rising temperatures prolong food availability and reduce thermoregulatory costs, hibernating species are generally expected to delay entry into hibernation and emerge earlier in spring. This pattern has been observed in bears (Delgado et al. 2018; Johnson et al. 2018). Similarly, Turbill and Prior (2016) found a negative correlation between mean annual temperature and hibernation duration in rodents. However, many fat‐storing rodents begin hibernation while environmental conditions are still favourable (e.g., Humphries et al. 2003), particularly when opportunities for reproduction are limited (e.g., European edible dormouse: Bieber et al. 2014; Hoelzl et al. 2015; Columbian ground squirrel: Neuhaus 2000). This behaviour is considered adaptive when the risks of remaining active outweigh the potential reproductive benefits, and when staying in the hibernaculum offers a survival advantage (Ruf and Bieber 2020; Constant et al. 2020). As a result, hibernation timing is likely to be state‐dependent and vary across sex and age classes within species (Allison et al. 2023). The sensitivity of hibernation phenology to climate change may therefore similarly vary significantly between and within species and remains difficult to predict. Tracking such changes requires long‐term, individualised data on hibernation phenology (e.g., Kerth and Wolf 2025). Unfortunately, such data on wild hibernators are difficult to obtain and therefore exist in only a few species (e.g., Cordes et al. 2020; Ozgul et al. 2010).

Bats represent one of the largest taxa capable of hibernation and differ from other hibernating mammals in several key aspects. For one, their reliance on flight impacts their ability to accumulate large fat reserves, compared to terrestrial hibernators whose locomotion costs are less affected by increased weight. Flight becomes exponentially more energy intensive as body weight increases, while concurrently manoeuvrability and the ability to catch prey declines (Willis 2017). Consequently, as bats start amassing fat reserves in autumn, further fat accumulation can only be achieved at progressively higher prey densities. Thus, the timing of bat hibernation phenology in autumn may be more directly linked to late season peaks in food availability than in other hibernating taxa. While most temperate‐zone bats are insectivorous, they vary considerably in terms of their dietary specialisation and foraging ecology (e.g., Russo and Fenton 2023). As a result, the point at which food availability becomes a constraining factor may differ markedly between species, leading to differences in hibernation phenology. For instance, bat species sharing the same hibernaculum have been found to differ in hibernation length by several months (Meier et al. 2022; Spitzenberger et al. 2023).

Bats also differ from many terrestrial hibernators in the seasonal timing of their reproductive investment. In temperate‐zone bat species, males invest heavily in spermatogenesis in late summer and early autumn, before hibernation, while females must invest more upon emergence in spring, when gestation begins (e.g., Norquay and Willis 2014). Consequently, female bats often enter hibernation with larger fat reserves than males (Jonasson and Willis 2011), and may be more susceptible to changes in prey phenology in spring. This contrasts with many rodents, in which both sexes invest in reproduction in spring (e.g., Willis 2017). However, in some rodents, males spread their reproductive efforts across both seasons by caching food and defending territories in autumn, and emerging early in spring to prepare for mating (e.g., arctic ground squirrels, Williams et al. 2016). Although several studies have documented shifts in the phenology of parturition and seasonal migration in bats (e.g., Frick et al. 2010; Matthäus et al. 2023; Stepanian and Wainwright 2018), there have been no long‐term, individualised studies investigating the extent to which bats alter their hibernation phenology in response to climate warming.

Our study focuses on the hibernation phenology of two temperate‐zone bat species, Daubenton's bat ( *Myotis daubentonii* ) and Natterer's bat ( *Myotis nattereri* ), at a hibernaculum in north‐western Germany. These two similarly sized species (6–15 g) occur throughout Europe, ranging from the Mediterranean to southern Scandinavia (Encarnação and Becker 2023; Razgour et al. 2023). Although they frequently hibernate together, their hibernation phenology differs markedly. *Myotis daubentonii* hibernates for approximately 6 months, twice as long as *M. nattereri* (Meier et al. 2022). This disparity is likely due to differences in their foraging ecology. *Myotis daubentonii* specialises in catching aquatic insects from the surface of water bodies (Jones and Rayner 1988), limiting its foraging ability in late autumn and early spring when aquatic insect emergence from water bodies is low (Salvarina et al. 2017). Conversely, *M. nattereri* is a generalist gleaner that can also catch non‐volant prey, such as spiders and caterpillars. Therefore, they can remain active at low ambient temperatures, when flying insect abundance is greatly reduced (Hope et al. 2014).

Significant differences in hibernation phenology have also been observed between the sexes and age classes of both investigated bat species. Adult males and juveniles tend to enter the hibernaculum later and emerge earlier than adult females (Meier et al. 2022). Moreover, as in other bat species (Kunz et al. 1998), juvenile bats of both species likely accumulate fat more slowly in autumn and enter hibernation at a lower body mass than adults. Adults of both species exhibit higher survival in winter than in summer, but the discrepancy in seasonal survival is more pronounced in *M. daubentonii* despite their longer hibernation duration (Reusch et al. 2019). These differences in foraging ecology and seasonal survival make these two species a compelling model for exploring how species‐specific ecological traits shape responses to climate change.

We examined species‐, sex‐ and age‐specific shifts in hibernation phenology across a dataset of several thousand RFID‐marked *M. daubentonii* and *M. nattereri* spanning 13 years (2010–2023) at a hibernaculum in Germany. Linear mixed models were used to quantify changes in hibernation start, end and duration over time. To evaluate whether these changes are temperature‐driven, we assessed the relationship between the hibernation start and end dates and the median air temperature in the 2 months preceding the respective event. We expected that warmer autumn and spring temperatures would extend the foraging period for both species, resulting in delayed entry and earlier emergence. The individual‐based nature of our data also allowed us to assess whether different sex and age classes within a species differed in their responses. Finally, based on our findings, we investigated the potential consequences of the observed phenological shifts in *M. daubentonii* by comparing body mass accumulation patterns during the annual capture periods (August–September) between the start and the end of the study period. We used these data to determine whether pre‐hibernation body mass had shifted in response to changes in hibernation phenology. In *M. nattereri* , fat accumulation typically begins after the period covered by our capture data (Kohyt et al. 2016) and therefore could not be assessed here.

## 2 Materials and Methods

The studied hibernaculum is a 60 m deep disused well shaft in north‐western North Rhine‐Westphalia, Germany. The well is surrounded by a house with a single entrance that is monitored by an infrared light barrier (Chirotec, Germany), which registers all bats entering and leaving the building (Krivek et al. 2023). During the study period (2010–2023), the annual hibernating population estimated from the light barrier data ranged between 5000 and 8000 bats. Seven bat species were identified through captures at the entrance of the well house, with *M. daubentonii* and *M. nattereri* being the most abundant, with several thousand individuals each.

The well shaft is covered by a wooden lid with three small entrances through which the bats must crawl to enter the hibernaculum (see Meier et al. 2024 for schematics). Since 2010, the two original entrances have been monitored with a single RFID reader. In 2015, a third entrance was created, and all three entrances were equipped with two RFID readers. The two readers within an entrance are spaced approximately 20 cm apart and are used to determine the direction of movement (i.e., entry or exit) based on the order in which the loggers were triggered by the passing bat. Note that although each entrance was fitted with a second reader in 2015, due to initial time synchronisation issues between the logging devices, directionality could only be determined from 2017 onwards.

### 2.1 Bat Capture

Starting in 2007, bats were captured in August and September using a 2‐bank harp trap designed to fit the dimensions of the entrance of the well house. Each year, up to 150 individuals of *M. daubentonii* (total *N* = 2214) and *M. nattereri* (total *N* = 2132) were tagged with RFID‐transponders (Trovan, 100 ID). For each individual, forearm length, body mass and sex were recorded, and age was classified as adult or juvenile based on epiphyseal closure, the presence/strength of their chin spot, dental calculus and tooth wear (Brunet‐Rossinni and Wilkinson 2009; Richardson 1994). The forearm length was measured to the nearest 0.1 mm using callipers, and the body mass was measured to the nearest 0.5 g using a digital scale. All bat captures, handling, marking and installations of monitoring systems were carried out under the license of the respective authorities (permit numbers: 50.0835.2.1, 84‐02.04.2015.A508, 70.2.2.27, 70.2‐0197/08, 70.2‐0228/10, 70.2‐2012/0254, 70.2‐2016/0023, 3139‐04, 67/5.1‐58/2008‐2019, 67 200 032).

### 2.2 RFID‐Monitoring and Data Handling

The activity of tagged bats at the well shaft was monitored throughout the year using RFID readers installed at all three entrances. To maintain coherence between the start and end of hibernation, a “bat year” was defined as spanning from 1 July to 30 June of the following year, with a “bat day” running from noon to noon. Juveniles were considered adults after their first year (as in Meier et al. 2024).

The dataset was divided into two periods: (1) from 2010 to 2017 (1 July 2010–30 June 2017), when only single RFID readers were used per entrance, and thus the directionality of the passes could not be determined; and (2) from 2017 to 2023 (1 July 2017–30 June 2023), when two RFID readers per entrance allowed the directionality of the passes of tagged bats to be assessed (i.e., entry or exit).

For both datasets, hibernation phenology was characterised for each individual using the longest hibernation period, defined as the longest interval without a recorded pass with a start date between 1 July and 1 February and an end date between 31 December and 30 April (as in Meier et al. 2022; sensu “ecological hibernation” Constant et al. 2024). For the directionality dataset (2017–2023), this interval was additionally required to start with an entry and end with an exit, and all cases where either entry or exit were not correctly recorded (e.g., one of the readers failed to record the individual's pass) were discarded. Power outages resulted in three data gaps: in March 2016 and in late November/December of both 2017 and 2018. As a result, the hibernation start for *M. nattereri* in 2017 and 2018, and hibernation end for both species in 2016 were excluded from the analysis (see Table S1 for further details).

To validate whether the two data collection methods yielded comparable results, the second half of the dataset was reanalysed as if it had been collected with a single RFID reader (i.e., using only data from the reader positioned closest to the reader location during the first half of the study). We found that hibernation end dates were identical between the two datasets in 98.8% of cases for *M. daubentonii* and 97.3% for *M. nattereri* , suggesting that the additional directionality filtering had a minimal effect on the determination of the start and end dates. We also used the directionality dataset to assess whether the observed end dates reliably reflected actual emergence from the hibernaculum by comparing the hibernation end date with the final departure date of the individual. The final departure date was defined as the first exit from the hibernaculum after the hibernation end date, which was not followed by an entry within 24 h. We found that hibernation end dates closely matched final departure dates, with the final departure occurring less than 1 week after the hibernation end date in 94.3% of cases for *M. daubentonii* and 92.2% for *M. nattereri* . These results support the use of the longest hibernation period as a reliable proxy for describing ecological hibernation phenology.

The final dataset comprised 2546 hibernation records from 948 individuals for *M. daubentonii* and 2343 records from 891 individuals for *M. nattereri* (see Table S1 for detailed sample sizes; Krivek et al. 2025). The dataset includes outliers that likely do not represent true entry or emergence dates: these can be caused by bats briefly exiting the well in mid‐winter, or when the true entry pass (i.e., hibernation start) was missed and an earlier entry was therefore considered as the hibernation start. To avoid introducing subjective bias, no additional filters were applied, and all data points were retained for modelling.

### 2.3 Statistical Analysis

All statistical analyses were performed in R (version 4.3.2; R Core Team 2023). Linear mixed models fitted with the *lme4* package (Bates et al. 2015) were used to examine temporal changes in the hibernation phenology of *M. daubentonii* and *M. nattereri* . Marginal effects of predictors were estimated using the *modelbased* package (Makowski et al. 2025). For each species, separate models were constructed for the hibernation start, end and duration. All models included “bat year” and a categorical variable summarizing sex and age (four categories: juvenile male, juvenile female, adult male, adult female) as fixed effects, as these factors are known to influence their hibernation phenology (Meier et al. 2022). In addition, the interaction between “bat year” and the sex‐age variable was included, as each class was expected to respond differently to climate change. Individual ID was included as a random effect in all models. Full model structures and summary outputs, including fixed and random effect estimates, are provided in Table S2.

We then investigated whether the observed shifts in hibernation start and end dates correlated with changes in ambient temperature in the period preceding the variable of interest, since ambient temperature has previously been shown to influence hibernation timing (Meyer et al. 2016). To do this, we obtained hourly air temperature data from a weather station in Münster, Germany, located approx. 20 km from the study site (Deutscherti Wetterdienst, Station ID 1766). For the models, we summarised temperature as the median air temperature for a 2‐month period prior to and including the variable of interest (i.e., median hibernation start or end date). We used median values to evaluate whether an overall warmer climate influences hibernation phenology, as it mitigates the impact of short but potentially extreme weather anomalies. To confirm that our choice of summary statistics did not affect the results, we examined the collinearity of the median, minimum and maximum temperature metrics. Median temperature was positively correlated with both maximum (*r* = 0.87–0.96 across periods) and minimum (*r* = 0.87–0.89) average daily temperatures. As these metrics were highly correlated, we retained median temperature as a parsimonious predictor in subsequent analyses. Since temperature‐driven insect availability in the months before entry and emergence might be crucial for hibernation timing, we selected a 2‐month window before median hibernation start and end dates as an indicator of potential foraging conditions for bats. For *M. daubentonii* , we used the median air temperature over August and September for the model of hibernation start, and over February and March for the model of hibernation end. For *M. nattereri* , the median air temperature over November and December was used for the model for hibernation start, and the median air temperature over January and February for the model for hibernation end (cf. Meier et al. 2022). All four models included the median air temperature, the sex and age class of the bat and an interaction between these two variables as fixed effects, and “bat year” as a random effect. Full model structures and summary outputs, including fixed and random effect estimates, are provided in Table S3.

### 2.4 Body Mass of *M. daubentonii* Over Time

Finally, we investigated the potential causes and consequences of the observed shift in hibernation start date in *M. daubentonii* by analysing body mass progression during the annual capture period (August–September) across sex and age classes. It is important to note that body mass data collection was usually stopped once the annual quota of individuals, allowed by the animal welfare permit, was reached. As a result, body mass data covering the entire 2‐month measurement window were only available for 3 years near the beginning of the study period (2011–2013) and for 1 year after the study period (2024). Using these data, we assessed whether body mass of *M. daubentonii* in autumn increased together with their extended hibernation duration.

## 3 Results

### 3.1 Hibernation Phenology Shift Over Time

**FIGURE 1:**
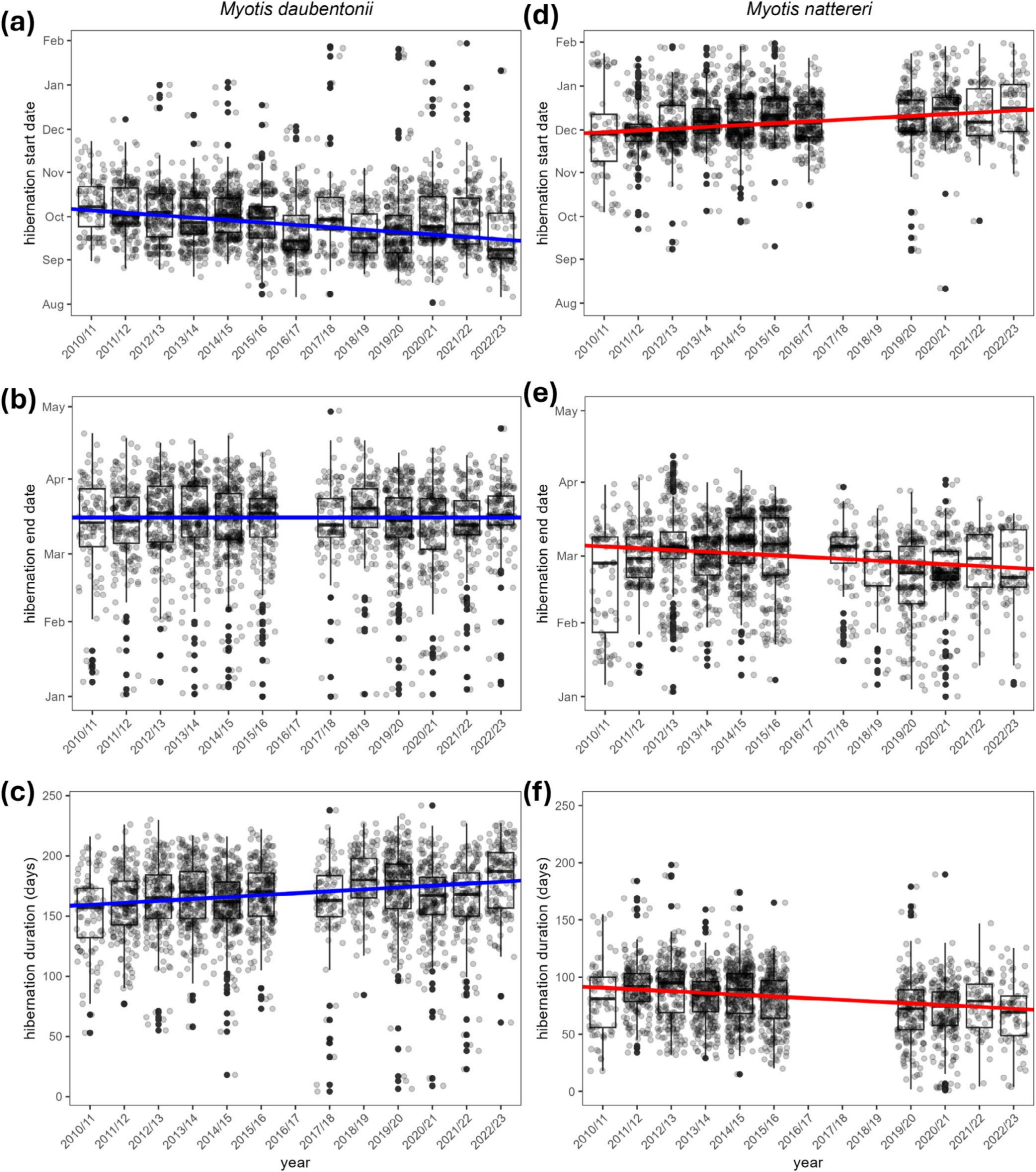
Hibernation phenology (hibernation start, end and duration) of two sympatric bat species over 13 years (2010/11–2022/23). (a) Hibernation start date, (b) end date, and (c) duration of *Myotis daubentonii* , and (d) hibernation start date, (e) end date, and (f) duration of *Myotis nattereri* . Linear regression lines show the overall trends of the median values. Power outages resulted in three data gaps, necessitating the exclusion of the 2016/17 season for analyses of hibernation end and duration in both species, as well as the exclusion of the 2017/18 and 2018/19 years for hibernation start and duration in *M. nattereri* .

**FIGURE 2:**
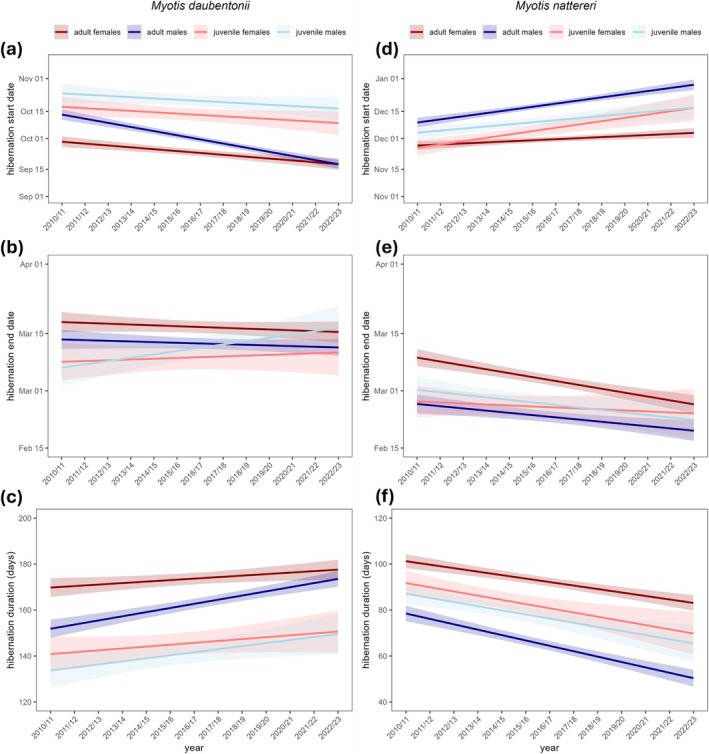
Sex‐ and age‐specific hibernation phenology of two sympatric bat species over 13 years (2010/11–2022/23) predicted with linear mixed models (95% confidence interval). (a) Hibernation start date, (b) end date, and (c) duration of *Myotis daubentonii* and (d) hibernation start date, (e) end date and (f) duration of *Myotis nattereri* .

During the 13‐year study, the start date of hibernation for *Myotis daubentonii* advanced by an average of 1.11 days/year, while the end date remained stable. This resulted in an increase in hibernation duration of almost 15 days over the study period (Figure 1a–c and Table 1). Adult males exhibited the most pronounced shift, with their hibernation start advancing by 2.14 days/year. This resulted in an annual increase of 1.81 days in hibernation duration, equating to a 23.53 day increase over the study period (Figure 2a,c). Adult females also entered the hibernaculum earlier by 0.97 days/year, extending their hibernation duration by 0.65 days/year (Figure 2a,c). Unlike both adult classes, neither juvenile class advanced its start date significantly. However, juvenile males significantly delayed their hibernation end date by 0.81 days/year, contributing to an overall increase in hibernation duration of 1.33 days/year (Figure 2b,c).

**TABLE 1:** Changes in the hibernation phenology of two sympatric bat species ( *Myotis daubentonii* and *M. nattereri* ) over time.

		Hibernation start date	Hibernation end date	Hibernation duration (days)
*Myotis daubentonii*	Adult females	**−0.97 days/year (*t* = −4.92, *p* < 0.001)**	−0.20 days/year (*t* = −1.17, *p* = 0.241)	**+0.65 days/year (*t* = 2.24, *p* = 0.025)**
	Adult males	**−2.14 days/year (*t* = −12.52, *p* < 0.001)**	−0.16 days/year (*t* = −1.07, *p* = 0.285)	**+1.81 days/year (*t* = 7.14, *p* < 0.001)**
	Juvenile females	−0.70 days/year (*t* = −1.75, *p* = 0.081)	+0.19 days/year (*t* = 0.54, *p* = 0.591)	+0.82 days/year (*t* = 1.39, *p* = 0.164)
	Juvenile males	−0.65 days/year (*t* = −1.66, *p* = 0.097)	**+0.81 days/year (*t* = 2.33, *p* = 0.020)**	**+1.33 days/year (*t* = 2.29, *p* = 0.022)**
	Overall	**−1.11 days/year (*t* = −6.97, *p* < 0.001)**	+0.16 days/year (*t* = 1.13, *p* = 0.258)	**+1.15 days/year (*t* = 4.90, *p* < 0.001)**
*Myotis nattereri*	Adult females	**+0.54 days/year (*t* = 3.28, *p* = 0.001)**	**−0.95 days/year (*t* = −6.26, *p* < 0.001)**	**−1.51 days/year (*t* = −6.84, *p* < 0.001)**
	Adult males	**+1.63 days/year (*t* = 8.70, *p* < 0.001)**	**−0.54 days/year (*t* = −3.19, *p* = 0.001)**	**−2.34 days/year (*t* = −9.44, *p* < 0.001)**
	Juvenile females	**+1.72 days/year (*t* = 4.27, *p* < 0.001)**	−0.24 days/year (*t* = −0.68, *p* = 0.499)	**−1.83 days/year (*t* = −3.45, *p* < 0.001)**
	Juvenile males	**+1.08 days/year (*t* = 3.21, *p* = 0.001)**	**−0.62 days/year (*t* = −2.06, *p* = 0.039)**	**−1.80 days/year (*t* = −3.99, *p* < 0.001)**
	Overall	**+1.24 days/year (*t* = 8.30, *p* < 0.001)**	**−0.59 day/year (*t* = −4.39, *p* < 0.001)**	**−1.87 days/year (*t* = −9.36, *p* < 0.001)**

*Note:* Species, sex‐ and age‐specific trends over 13 years (2010/11–2022/23) are based on linear mixed models. Coefficients represent the estimated marginal effects per year on the hibernation start date, end date and duration within each sex‐age class and across all four subclasses (‘overall’). Statistically significant results (*p* < 0.05) are shown in bold.

*Myotis nattereri* showed an opposite response, delaying its start of hibernation by 1.24 days/year and advancing its end by 0.59 days/year. These changes resulted in an overall decrease in hibernation duration of 24.31 days over the study period (Figure 1d–f and Table 1). This pattern was consistent across all sex‐age classes, with the start of hibernation shifting later in all classes (range: 0.54–1.72 days/year; Figure 2d and Table 1). Similarly, the end of hibernation advanced in all classes (from 0.24 to 0.95 days/year), although this shift was not significant in juvenile females (Figure 2e and Table 1). Hibernation duration concordantly decreased significantly in all classes. The largest reduction was observed in adult males, where hibernation duration decreased by more than 30 days over the study period (range: 1.51–2.34 days/year; Figure 2f and Table 1).

### 3.2 Effect of Median Air Temperature on Hibernation Phenology

**FIGURE 3:**
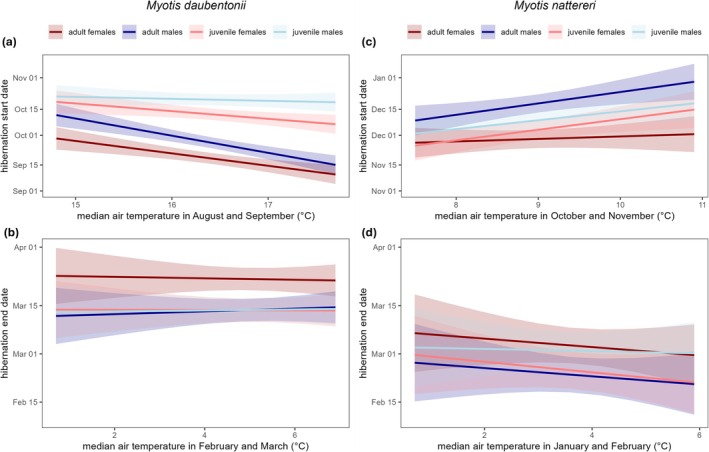
Sex‐ and age‐specific hibernation phenology of two sympatric bat species in relation to the median air temperature in a two‐month window before the start and end of their hibernation period over 13 years (2010/11–2022/23). Trends are predicted with linear mixed models with a 95% confidence interval. (a) Median hibernation start date of *Myotis daubentonii* and median air temperature in August and September, (b) median hibernation end date of *M. daubentonii* and median air temperature in February and March, (c) median hibernation start date *of Myotis nattereri* and median air temperature in October and November and (d) median hibernation end date of *M. nattereri* and median air temperature in January and February.

When analysing the effect of median air temperature over a 2‐month period prior to the median start and end dates of the hibernation period, we observed a significant relationship between temperature and start date of hibernation in most sex‐age classes of both species (Table 2 and Figure 3).

**TABLE 2:** Changes in the median hibernation start and end dates of two sympatric bat species ( *Myotis daubentonii* and *M. nattereri* ) in relation to the median air temperature in a two‐month window around their species‐specific hibernation start and end dates (in August/September and February/March for *M. daubentonii* , and in November/December and January/February for *M. nattereri* ).

		Median hibernation start date	Median hibernation end date
*Myotis daubentonii*	Adult females	**−6.69 days (*t* = −4.15, *p* < 0.001)**	−0.21 days (*t* = −0.25, *p* = 0.807)
	Adult males	**−9.25 days (*t* = −5.74, *p* < 0.001)**	−0.40 days (*t* = 0.47, *p* = 0.643)
	Juvenile females	**−4.15 days (*t* = −2.56, *p* = 0.015)**	−0.04 days (*t* = −0.05, *p* = 0.963)
	Juvenile males	−1.07 days (*t* = −0.66, *p* = 0.512)	+0.15 days (*t* = 0.18, *p* = 0.861)
*Myotis nattereri*	Adult females	+1.35 days (*t* = 0.64, *p* = 0.529)	−1.23 days (*t* = −0.75, *p* = 0.460)
	Adult males	**+6.15 days (*t* = 2.90, *p* = 0.007)**	−1.20 days (*t* = −0.73, *p* = 0.471)
	Juvenile females	**+5.71 days (*t* = 2.67, *p* = 0.013)**	−1.53 days (*t* = −0.94, *p* = 0.356)
	Juvenile males	**+4.79 days (*t* = 2.24, *p* = 0.033)**	−0.32 days (*t* = −0.19, *p* = 0.848)

*Note:* Sex‐ and age‐specific trends over 13 years (2010/11–2022/23) are based on linear mixed models. Coefficients represent the estimated marginal effects of a 1°C increase in median temperature on the median hibernation start and end dates. Statistically significant results (*p* < 0.05) are shown in bold.

In *M. daubentonii* , both adult classes and juvenile females advanced their median hibernation start date in response to an increase in median air temperature in August and September. Adult males advanced their entry into the hibernaculum by over 9 days for every 1°C increase in median air temperature (Table 2). However, median hibernation end date was not significantly correlated with median temperatures in February and March for any class.

For *M. nattereri* , median hibernation start date was significantly positively correlated with increasing median air temperatures in November and December for adult males, juvenile males and juvenile females (Table 2), but not for adult females. There was no significant correlation between median hibernation end date and median air temperature in January and February, although a negative trend (> −1 day/°C) was observed in three of the four sex‐age classes.

### 3.3 Body Mass of *Myotis daubentonii* Over Time

**FIGURE 4:**
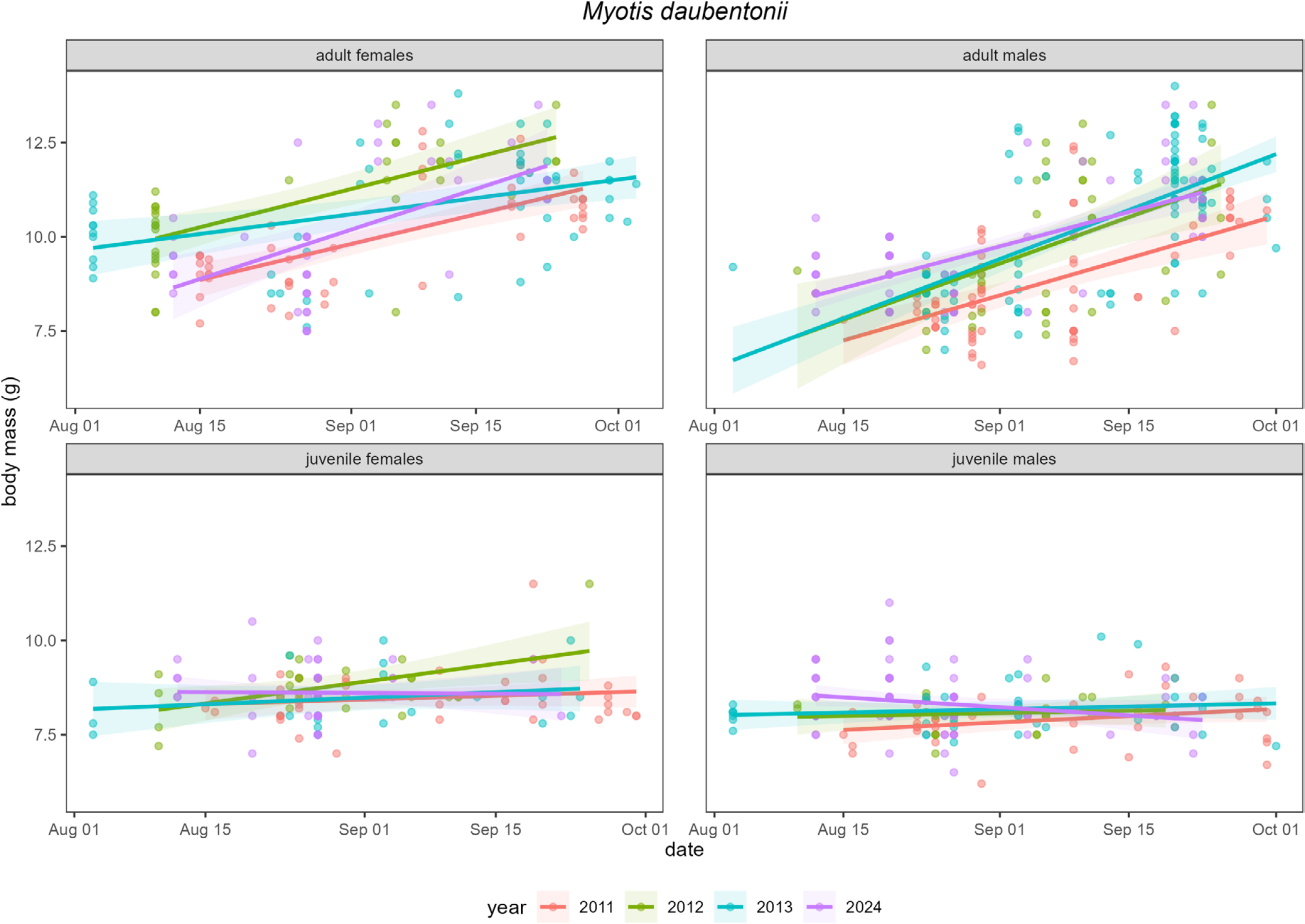
Pre‐hibernation body mass of *Myotis daubentonii* individuals captured between 1 August and 1 October in 2011–2013 and 2024, categorised by sex and age class.

Based on the available dataset, seasonal body mass gains prior to hibernation remained broadly consistent across years in all sex and age classes. Despite their extended hibernation duration, there was no evidence that individuals had higher body masses in the pre‐hibernation period in 2024 than in previous years (Figure 4). Strikingly, while body mass increased substantially in both adult classes over August and September as expected, both juvenile classes showed little to no increase in body mass during this time (Figure 4).

## 4 Discussion

Over the course of the 13‐year study period, we observed rapid but opposing shifts in the hibernation phenology of two sympatric bat species that correlated with warming temperatures. As expected, *Myotis nattereri* shortened its hibernation duration by delaying entry and advancing emergence, presumably because warmer temperatures increase prey availability in late autumn and early spring. However, *M. daubentonii* unexpectedly extended its hibernation duration by entering the hibernaculum earlier. To our knowledge, this is the first documented case of a species extending its hibernation duration in response to warming climate. The contrasting trends observed in these two species were primarily driven by changes in hibernation entry in autumn, highlighting the importance of this frequently overlooked season (Gallinat et al. 2015).

We also observed notable differences in phenological shifts across sex and age classes within both species, with adult males showing the largest changes. In *M. daubentonii* , adult males extended their hibernation duration by nearly one month over the 13‐year study period (1.81 days/year). In *M. nattereri* , adult males reduced their hibernation duration by over 1 month (2.34 days/year), resulting in a one‐third reduction in total hibernation duration. These rates are truly remarkable, exceeding those reported for other hibernating mammals (e.g., Chmura et al. 2023; Inouye et al. 2000; Lane et al. 2012), and may have significant implications for energy balance, winter survival and long‐term population dynamics.

### 4.1 *Myotis daubentonii*

In autumn, both adult male and female *M. daubentonii* significantly advanced their entry into the hibernaculum. These shifts were strongly negatively correlated with median air temperatures in August and September, suggesting a temperature‐sensitive phenological response. We hypothesise that the effect of temperature is indirect and that the observed advancement of the entry time likely reflects a response to changes in the availability of aquatic insects, which are the species' primary prey. Insect emergence from water bodies is highly temperature‐dependent and typically occurs in synchronised peaks (Salvarina et al. 2017). In response to climate change, the timing of this emergence has shifted. Baranov et al. (2020) observed that the emergence peak has become more diffuse and has advanced by 13.4 days over the course of a 42‐year study, though several taxa exhibited contrasting patterns. While specific emergence data on the prey species of *M. daubentonii* are lacking, it is plausible that the abundance of key aquatic insect prey is declining in autumn. The earlier entry into hibernation of *M. daubentonii* may therefore represent a behavioural adjustment to shifting prey availability. In addition, as *M. daubentonii* exhibits higher seasonal survival rates in winter than in summer (Reusch et al. 2019), individuals may benefit from entering the hibernaculum as early as possible, once they have accumulated sufficient fat reserves. Similar patterns have been observed in other hibernators (e.g., golden‐mantled ground squirrels), where later entry was linked to increased mortality risk (Vallance et al. 2025).

Notably, juveniles of both sexes did not exhibit a significant shift in entry phenology. As a result, juvenile *M. daubentonii* now enter the hibernaculum approximately a month later than adults. Moreover, juvenile body mass did not increase substantially over the course of August and September, as it did in adults. This suggests that juveniles may be unable to accumulate fat reserves as efficiently as adults, possibly because their foraging success is lower than that of adults.

While the timing of entry into hibernation has advanced in correlation with warmer autumn temperatures, the timing of the emergence has remained stable and showed no correlation with spring temperatures. Circannual timing in some hibernators is photoperiodic, while in other species it is regulated by an endogenous clock which can be synchronised with external cues (Williams et al. 2014). In bats, as in other hibernators (e.g., Evans et al. 2016), emergence from hibernation may be primarily driven by internal physiological mechanisms—such as circannual rhythms (Helm et al. 2013)—rather than by external conditions. We speculate that this may result in a phenological mismatch, given that the spring emergence peak of many aquatic insects has advanced in response to climate change (e.g., Baranov et al. 2020; Marshall et al. 2020). However, studies investigating insect availability and bat foraging success in early spring are currently lacking.

Based on our body mass data from August and September, the observed increase in hibernation duration does not appear to be accompanied by an increase in pre‐hibernation mass. This suggests that individuals are relying on similar fat reserves as before to sustain them for a longer hibernation period. Since torpor is associated with considerable costs, such as increased oxidative stress and suppressed immune function, extending torpor bout length and depth in order to compensate for lower fat reserves will result in higher physiological costs (e.g., Boyles et al. 2020; Humphries et al. 2003). Alternatively, if individuals do not adjust their torpor expression, they are likely to emerge from hibernation in poorer body condition, which will affect their survival and reproductive investment in spring.

Finally, the observed shifts in hibernation phenology in *M. daubentonii* may also affect their mating system. Adult males exhibited the fastest advance in hibernation entry, resulting in a convergence in entry timing between the sexes, which had previously differed by several weeks (Meier et al. 2022). Males of this species show two distinct mating strategies: dominant males mate with females in the summer habitat (Encarnaçao 2012; Senior et al. 2005), while further mating occurs during autumn swarming at hibernacula (e.g., van Schaik et al. 2015). Swarming is thought to be energetically expensive, and males typically delay fat accumulation until females have entered hibernation (e.g., Kohyt et al. 2016). Therefore, the convergence in hibernation entry phenology may signal a shift in mating dynamics, with fewer males engaging in costly swarming and more entering the hibernaculum alongside females. A similar divergence in the phenological response of males and females to warming temperatures has been observed in arctic ground squirrels (Chmura et al. 2023), in which females advanced their emergence from hibernation but males did not.

Taken together, the magnitude of the observed shift in the hibernation phenology of *M. daubentonii* raises concerns about the long‐term viability of the population. First, the current rate at which entry into the hibernaculum is advancing does not seem to be sustainable, as the duration of hibernation cannot increase indefinitely given that the accumulation of fat reserves is limited by the need to fly (e.g., Willis 2017). If earlier entry becomes necessary due to continued advances in insect phenology, *M. daubentonii* may approach the physiological limits of its energy budget during hibernation. Given the rapid pace of the observed phenological shifts, it also seems plausible that, once these responses reach their limits, there could be an abrupt change in their survival. Second, if hibernation entry continues to advance, it will encroach upon the autumn mating period and the summer maternity phase, unless these behaviours also advance (Lučan et al. 2013; Mundinger et al. 2022). Although individual variation in strategies may offer some resilience, the population as a whole will either need to adjust its annual cycle in new ways or face the risk of maladaptive outcomes, with potentially dramatic demographic consequences. Finally, if juveniles are unable to adjust their hibernation timing, 1st‐year survival is likely to decline. This could ultimately undermine the long‐term viability of the population.

### 4.2 *Myotis nattereri*

During the study period, all sexes and age classes of *M. nattereri* delayed their entry into and advanced their emergence from the hibernaculum. This resulted in an overall reduction in hibernation duration of up to 2.34 days/year. The shift in hibernation entry correlated significantly with median ambient temperature in November and December for adult males and both juvenile classes. The reduction in hibernation duration may be driven by *M. nattereri* 's flexible foraging ecology, particularly its ability to catch non‐volant prey even at low ambient temperatures (Hope et al. 2014). This enables the species to exploit the extended foraging opportunities associated with warming autumns and winters—a strategy also observed in other hibernators (Bieber et al. 2014; Johnson et al. 2018). Furthermore, as summer survival of *M. nattereri* is higher than in *M. daubentonii* (Reusch et al. 2019), the relative mortality risk of remaining active versus entering the hibernaculum may be lower. Consequently, individuals may benefit from delaying entry into the hibernaculum until environmental conditions force them to hibernate.

While the overall patterns were similar for both sexes, males showed larger shifts in entry timing, whereas females showed larger shifts in emergence timing. These findings support the “thrifty female, frisky male” hypothesis, which suggests that males benefit most from increased investment in mating during autumn before starting fat accumulation, whereas females prioritise entering hibernation with greater fat reserves and may benefit most from an earlier emergence to prepare for gestation (Czenze et al. 2017; Jonasson and Willis 2011). Earlier emergence from hibernation has been linked to earlier parturition in other bat species (Matthäus et al. 2023; Stepanian and Wainwright 2018), which in turn enables a greater proportion of juveniles to attempt to reproduce in their first year (Frick et al. 2010; Linton and Macdonald 2020).

Unlike *M. daubentonii* , both juvenile classes of *M. nattereri* exhibited similar shifts in hibernation timing to adults, indicating a consistent response across age groups. This may be because of the longer period between birth dates in summer and hibernation entry in this species, which gives juveniles a longer period to complete skeletal growth and become proficient foragers before hibernating.

If the observed phenological shifts continue at their current rate, *M. nattereri* could become active year‐round within decades. However, this seems unlikely since, even under favourable conditions, hibernators generally do not avoid torpor entirely (Boyles et al. 2020; Humphries et al. 2003). A more likely scenario is that hibernation will become fragmented, with individuals emerging from their hibernaculum throughout mid‐winter to forage on favourable nights, as has been observed in *Pipistrellus pipistrellus* (Avery 1985). Additionally, climate change is also expected to increase weather stochasticity and the frequency of extreme weather events, which could in turn increase the occurrence of mass mortality events and population declines (Reusch et al. 2019).

### 4.3 Lessons for Other Hibernators

Our findings challenge the common assumption that climate change generally leads to shorter hibernation periods by demonstrating that even sympatrically hibernating species can exhibit strikingly divergent responses to climate change in terms of their phenology. Although the rapid advancement of hibernation entry observed in *M. daubentonii* in correlation with climate change is currently the only documented example of such a shift, such advancements may be more widespread in hibernators with specialised diets. The annual phenology of many plants and arthropods has been shown to advance in response to rising temperatures (e.g., Forrest 2016; Piao et al. 2019). As a result, food resources with a single annual peak, such as many fruiting plants or arthropods that complete one generation per year (i.e., univoltine insects), may be reduced in autumn (Gallinat et al. 2015). Hibernators that rely on such food resources to accumulate fat before hibernation may therefore need to enter hibernation earlier to avoid late‐season food scarcity.

Our data also highlight the importance of long‐term, individual‐level monitoring of hibernation phenology (as discussed in Wells et al. 2022; Findlay‐Robinson et al. 2023; Kerth and Wolf 2025). The timing of hibernation represents a complex trade‐off between energy conservation, mortality risk and reproductive investment. This trade‐off is influenced by both extrinsic factors, such as food availability, predation and ambient temperature, as well as intrinsic constraints, such as fat reserves and reproductive status (Allison et al. 2023; Constant et al. 2020). Therefore, population‐level studies alone may overlook the fine‐scale selection pressures that shape individual responses to climate change.

## 5 Conclusion and Outlook

Our findings provide clear evidence that the hibernation phenology of two temperate‐zone bat species has changed rapidly with climate change. As temperatures continue to rise, the contrasting hibernation strategies observed here could have a significant impact on the long‐term survival of the species studied. However, the direct links between these shifts in phenology and individual fitness or population‐level outcomes remain to be established. Further research is particularly needed to investigate how these shifts in hibernation phenology influence energy expenditure and torpor expression throughout the hibernation phase (Boyles et al. 2020; Czenze et al. 2017; Humphries et al. 2003; Willis 2017). While our individual‐level monitoring of over 4000 hibernation events demonstrates that bats can exhibit rapid phenological shifts, it is unclear whether similar responses occur in other populations or species. Individualised studies across the range of these and other bat species are therefore urgently needed. Although tracking large numbers of tagged individuals is challenging, our study showcases that such long‐term monitoring of individuals at their hibernaculum is feasible.

Our results also have important implications for applied conservation. For example, in Germany access to hibernacula is restricted between 1 October and 31 March to protect hibernating bats (Bundesnaturschutzgesetz, Bundesgesetzblatt I, 2542 2009/2024, § 39 Abs. 6). However, this period no longer covers the entry of *M. daubentonii* and may therefore need to be revised. Conversely, the extended active period of *M. nattereri* in autumn and spring should be considered in forest management practices within their summer habitats, where bats are often assumed to be absent in winter.

## Conflicts of Interest

The authors declare no conflicts of interest.

## Supporting information


**Tables S1–S3:** gcb70531‐sup‐0001‐TableS1‐S3.pdf.

## Data Availability

The data that support the findings of this study are openly available in Zenodo at https://doi.org/10.5281/zenodo.15867838.

## References

[gcb70531-bib-0001] Allison, A. Z. , C. J. Conway , and A. E. Morris . 2023. “Why Hibernate? Tests of Four Hypotheses to Explain Intraspecific Variation in Hibernation Phenology.” Functional Ecology 37, no. 6: 1580–1593.

[gcb70531-bib-0002] Avery, M. I. 1985. “Winter Activity of Pipistrelle Bats.” Journal of Animal Ecology 54: 721–738.

[gcb70531-bib-0003] Baranov, V. , J. Jourdan , F. Pilotto , R. Wagner , and P. Haase . 2020. “Complex and Nonlinear Climate‐Driven Changes in Freshwater Insect Communities Over 42 Years.” Conservation Biology 34, no. 5: 1241–1251. 32022305 10.1111/cobi.13477

[gcb70531-bib-0004] Bates, D. , M. Mächler , B. Bolker , and S. Walker . 2015. “Fitting Linear Mixed‐Effects Models Using lme4.” Journal of Statistical Software 67: 1–48.

[gcb70531-bib-0005] Bieber, C. , K. Lebl , G. Stalder , F. Geiser , and T. Ruf . 2014. “Body Mass Dependent Use of Hibernation: Why Not Prolong the Active Season, If They Can?” Functional Ecology 28, no. 1: 167–177.

[gcb70531-bib-0006] Boyles, J. G. , V. Brack Jr. , K. E. Marshall , and D. Brack . 2024. “Shifts in Population Density Centers of a Hibernating Mammal Driven by Conflicting Effects of Climate Change and Disease.” Global Change Biology 30, no. 1: e17035. 37987538 10.1111/gcb.17035

[gcb70531-bib-0007] Boyles, J. G. , J. S. Johnson , A. Blomberg , and T. M. Lilley . 2020. “Optimal Hibernation Theory.” Mammal Review 50, no. 1: 91–100.

[gcb70531-bib-0008] Brunet‐Rossinni, A. , and G. S. Wilkinson . 2009. “Methods for Age Estimation and the Study of Senescence in Bats.” In Ecological and Behavioral Methods for the Study of Bats, 315–325. Johns Hopkins University Press.

[gcb70531-bib-0009] Bundesnaturschutzgesetz, Bundesgesetzblatt I, 2542 . 2009. “Last Amended by Art. 48 Gesetz of 23 October 2024, Bundesgesetzblatt I Nr. 323.” https://www.gesetze‐im‐internet.de/bnatschg_2009/.

[gcb70531-bib-0010] Chmura, H. E. , C. Duncan , G. Burrell , B. M. Barnes , C. L. Buck , and C. T. Williams . 2023. “Climate Change Is Altering the Physiology and Phenology of an Arctic Hibernator.” Science 380, no. 6647: 846–849. 37228197 10.1126/science.adf5341

[gcb70531-bib-0011] Constant, T. , F. S. Dobson , C. Habold , and S. Giroud . 2024. “Evolutionary Trade‐Offs in Dormancy Phenology.” eLife 12: RP89644. 38669069 10.7554/eLife.89644 PMC11052570

[gcb70531-bib-0012] Constant, T. , S. Giroud , V. A. Viblanc , et al. 2020. “Integrating Mortality Risk and the Adaptiveness of Hibernation.” Frontiers in Physiology 11: 706. 32754044 10.3389/fphys.2020.00706 PMC7366871

[gcb70531-bib-0013] Cordes, L. S. , D. T. Blumstein , K. B. Armitage , et al. 2020. “Contrasting Effects of Climate Change on Seasonal Survival of a Hibernating Mammal.” Proceedings of the National Academy of Sciences of the United States of America 117, no. 30: 18119–18126. 32631981 10.1073/pnas.1918584117 PMC7395557

[gcb70531-bib-0015] Czenze, Z. J. , K. A. Jonasson , and C. K. Willis . 2017. “Thrifty Females, Frisky Males: Winter Energetics of Hibernating Bats From a Cold Climate.” Physiological and Biochemical Zoology 90, no. 4: 502–511. 28641050 10.1086/692623

[gcb70531-bib-0016] Delgado, M. , G. Tikhonov , E. Meyke , et al. 2018. “The Seasonal Sensitivity of Brown Bear Denning Phenology in Response to Climatic Variability.” Frontiers in Zoology 15: 1–11. 30410564 10.1186/s12983-018-0286-5 PMC6211405

[gcb70531-bib-0017] Encarnaçao, J. A. 2012. “Mating at Summer Sites: Indications From Parentage Analysis and Roosting Behaviour of Daubenton's Bats ( *Myotis daubentonii* ).” Conservation Genetics 13: 1161–1165.

[gcb70531-bib-0018] Encarnação, J. A. , and N. I. Becker . 2023. “Daubenton's Bat *Myotis daubentonii* (Kuhl, 1817).” In Chiroptera, 225–255. Springer International Publishing.

[gcb70531-bib-0019] Evans, A. L. , N. J. Singh , A. Friebe , et al. 2016. “Drivers of Hibernation in the Brown Bear.” Frontiers in Zoology 13: 1–14. 26870151 10.1186/s12983-016-0140-6 PMC4750243

[gcb70531-bib-0020] Findlay‐Robinson, R. , V. B. Deecke , A. Weatherall , and D. L. Hill . 2023. “Effects of Climate Change on Life‐History Traits in Hibernating Mammals.” Mammal Review 53, no. 2: 84–98.

[gcb70531-bib-0021] Forrest, J. R. 2016. “Complex Responses of Insect Phenology to Climate Change.” Current Opinion in Insect Science 17: 49–54. 27720073 10.1016/j.cois.2016.07.002

[gcb70531-bib-0022] Frick, W. F. , D. S. Reynolds , and T. H. Kunz . 2010. “Influence of Climate and Reproductive Timing on Demography of Little Brown *Myotis myotis lucifugus* .” Journal of Animal Ecology 79, no. 1: 128–136. 19747346 10.1111/j.1365-2656.2009.01615.x

[gcb70531-bib-0023] Gallinat, A. S. , R. B. Primack , and D. L. Wagner . 2015. “Autumn, the Neglected Season in Climate Change Research.” Trends in Ecology & Evolution 30, no. 3: 169–176. 25662784 10.1016/j.tree.2015.01.004

[gcb70531-bib-0024] Geiser, F. 2021. Ecological Physiology of Daily Torpor and Hibernation. Vol. 317. Springer.

[gcb70531-bib-0025] Helm, B. , R. Ben‐Shlomo , M. J. Sheriff , et al. 2013. “Annual Rhythms That Underlie Phenology: Biological Time‐Keeping Meets Environmental Change.” Proceedings of the Royal Society B: Biological Sciences 280, no. 1765: 20130016. 10.1098/rspb.2013.0016 PMC3712433 23825201

[gcb70531-bib-0026] Hoelzl, F. , C. Bieber , J. S. Cornils , et al. 2015. “How to Spend the Summer? Free‐Living Dormice ( *Glis glis* ) Can Hibernate for 11 Months in Non‐Reproductive Years.” Journal of Comparative Physiology B 185, no. 8: 931–939. 10.1007/s00360-015-0929-1 PMC4628641 26293446

[gcb70531-bib-0027] Hope, P. R. , K. Bohmann , M. T. P. Gilbert , M. L. Zepeda‐Mendoza , O. Razgour , and G. Jones . 2014. “Second Generation Sequencing and Morphological Faecal Analysis Reveal Unexpected Foraging Behaviour by *Myotis nattereri* (Chiroptera, Vespertilionidae) in Winter.” Frontiers in Zoology 11: 1–15. 25093034 10.1186/1742-9994-11-39 PMC4108090

[gcb70531-bib-0028] Humphries, M. M. , D. W. Thomas , and D. L. Kramer . 2003. “The Role of Energy Availability in Mammalian Hibernation: A Cost‐Benefit Approach.” Physiological and Biochemical Zoology 76, no. 2: 165–179. 12794670 10.1086/367950

[gcb70531-bib-0029] Inouye, D. W. , B. Barr , K. B. Armitage , and B. D. Inouye . 2000. “Climate Change Is Affecting Altitudinal Migrants and Hibernating Species.” Proceedings of the National Academy of Sciences 97, no. 4: 1630–1633. 10.1073/pnas.97.4.1630 PMC26486 10677510

[gcb70531-bib-0030] Johnson, H. E. , D. L. Lewis , T. L. Verzuh , et al. 2018. “Human Development and Climate Affect Hibernation in a Large Carnivore With Implications for Human–Carnivore Conflicts.” Journal of Applied Ecology 55, no. 2: 663–672.

[gcb70531-bib-0031] Jonasson, K. A. , and C. K. Willis . 2011. “Changes in Body Condition of Hibernating Bats Support the Thrifty Female Hypothesis and Predict Consequences for Populations With White‐Nose Syndrome.” PLoS One 6, no. 6: e21061. 21731647 10.1371/journal.pone.0021061 PMC3120823

[gcb70531-bib-0032] Jones, G. , and J. Rayner . 1988. “Flight Performance, Foraging Tactics and Echolocation in Free‐Living Daubenton's Bats *Myotis daubentonii* (Chiroptera: Vespertilionidae).” Journal of Zoology 215, no. 1: 113–132.

[gcb70531-bib-0033] Kerth, G. , and J. M. Wolf . 2025. “In‐Situ Responses of Temperate‐Zone Bats to Climate Change.” Annals of the New York Academy of Sciences 1546, no. 1: 23–34. 40112255 10.1111/nyas.15317 PMC11998482

[gcb70531-bib-0034] Kohyt, J. , A. Rozik , K. Kozakiewicz , A. Pereswiet‐Soltan , and W. J. Gubała . 2016. “Activity Pattern and Fat Accumulation Strategy of the Natterer's Bat (Vespertilionidae, Chiroptera) Swarming Population Indicate the Exact Time of Male Mating Effort.” Mammal Research 61: 383–389.

[gcb70531-bib-0035] Krivek, G. , E. Mahecha , F. Meier , G. Kerth , and J. van Schaik . 2023. “Counting in the Dark: Estimating Population Size and Trends of Bat Assemblages at Hibernacula Using Infrared Light Barriers.” Animal Conservation 26, no. 5: 701–713.

[gcb70531-bib-0036] Krivek, G. , F. Meier , L. Grosche , G. Kerth , and J. van Schaik . 2025. “Dataset: One Species Hibernates Shorter, the Other Longer: Rapid but Opposing Responses to Warming Climate in Two Sympatric Bat Species.” *Zenodo*. 10.5281/zenodo.15867838.41039796

[gcb70531-bib-0037] Kunz, T. H. , J. A. Wrazen , and C. D. Burnett . 1998. “Changes in Body Mass and Fat Reserves in Pre‐Hibernating Little Brown Bats ( *Myotis lucifugus* ).” Ecoscience 5, no. 1: 8–17.

[gcb70531-bib-0038] Lane, J. E. , L. E. Kruuk , A. Charmantier , J. O. Murie , and F. S. Dobson . 2012. “Delayed Phenology and Reduced Fitness Associated With Climate Change in a Wild Hibernator.” Nature 489, no. 7417: 554–557. 22878721 10.1038/nature11335

[gcb70531-bib-0039] Linton, D. M. , and D. W. Macdonald . 2020. “Phenology of Reproductive Condition Varies With Age and Spring Weather Conditions in Male *Myotis daubentonii* and *M. nattereri* (Chiroptera: Vespertilionidae).” Scientific Reports 10, no. 1: 6664. 32313091 10.1038/s41598-020-63538-y PMC7171103

[gcb70531-bib-0040] Lučan, R. , M. Weiser , and V. Hanák . 2013. “Contrasting Effects of Climate Change on the Timing of Reproduction and Reproductive Success of a Temperate Insectivorous Bat.” Journal of Zoology 290, no. 2: 151–159.

[gcb70531-bib-0041] Makowski, D. , M. S. Ben‐Shachar , B. M. Wiernik , I. Patil , R. Thériault , and D. Lüdecke . 2025. “Modelbased: An R Package to Make the Most Out of Your Statistical Models Through Marginal Means, Marginal Effects, and Model Predictions.” Journal of Open Source Software 10, no. 109: 7969.

[gcb70531-bib-0042] Marshall, K. E. , K. Gotthard , and C. M. Williams . 2020. “Evolutionary Impacts of Winter Climate Change on Insects.” Current Opinion in Insect Science 41: 54–62. 32711362 10.1016/j.cois.2020.06.003

[gcb70531-bib-0043] Matthäus, L. , K. Kugelschafter , and J. Fietz . 2023. “Influence of Ambient Temperature on the Phenology of the Greater Mouse‐Eared Bat (*Myotis myotis*).” Ecology and Evolution 13, no. 5: e10081. 37214612 10.1002/ece3.10081 PMC10196220

[gcb70531-bib-0044] Meier, F. , L. Grosche , G. Krivek , et al. 2024. “Automated Long‐Term Monitoring of RFID‐Tagged Individuals Reveals High Hibernaculum Site Fidelity in Daubenton's Bats and Natterer's Bats.” Animal Conservation 28: 401–409.

[gcb70531-bib-0045] Meier, F. , L. Grosche , C. Reusch , V. Runkel , J. van Schaik , and G. Kerth . 2022. “Long‐Term Individualized Monitoring of Sympatric Bat Species Reveals Distinct Species‐and Demographic Differences in Hibernation Phenology.” BMC Ecology and Evolution 22, no. 1: 1–12. 35090401 10.1186/s12862-022-01962-6 PMC8796590

[gcb70531-bib-0046] Meyer, G. A. , J. A. Senulis , and J. A. Reinartz . 2016. “Effects of Temperature and Availability of Insect Prey on Bat Emergence From Hibernation in Spring.” Journal of Mammalogy 97, no. 6: 1623–1633.

[gcb70531-bib-0047] Mundinger, C. , T. Fleischer , A. Scheuerlein , and G. Kerth . 2022. “Global Warming Leads to Larger Bats With a Faster Life History Pace in the Long‐Lived Bechstein's Bat ( *Myotis bechsteinii* ).” Communications Biology 5, no. 1: 682. 35810175 10.1038/s42003-022-03611-6 PMC9271042

[gcb70531-bib-0048] Neuhaus, P. 2000. “Timing of Hibernation and Molt in Female Columbian Ground Squirrels.” Journal of Mammalogy 81, no. 2: 571–577.

[gcb70531-bib-0049] Norquay, K. , and C. Willis . 2014. “Hibernation Phenology of *Myotis lucifugus* .” Journal of Zoology 294, no. 2: 85–92.

[gcb70531-bib-0050] Ozgul, A. , D. Z. Childs , M. K. Oli , et al. 2010. “Coupled Dynamics of Body Mass and Population Growth in Response to Environmental Change.” Nature 466, no. 7305: 482–485. 20651690 10.1038/nature09210 PMC5677226

[gcb70531-bib-0051] Parmesan, C. , and G. Yohe . 2003. “A Globally Coherent Fingerprint of Climate Change Impacts Across Natural Systems.” Nature 421, no. 6918: 37–42. 12511946 10.1038/nature01286

[gcb70531-bib-0052] Piao, S. , Q. Liu , A. Chen , et al. 2019. “Plant Phenology and Global Climate Change: Current Progresses and Challenges.” Global Change Biology 25, no. 6: 1922–1940. 30884039 10.1111/gcb.14619

[gcb70531-bib-0053] R Core Team . 2023. “R: A Language and Environment for Statistical Computing.” R Foundation for Statistical Computing. https://www.R‐project.org/.

[gcb70531-bib-0054] Razgour, O. , C. Ibáñez , S. J. Puechmaille , and J. Juste . 2023. “ *Myotis nattereri* Species Complex ( *M. nattereri* , *M. crypticus*, and *M. escalerai* ).” In Handbook of the Mammals of Europe, 1–41. Springer International Publishing.

[gcb70531-bib-0055] Reusch, C. , J. Gampe , A. Scheuerlein , F. Meier , L. Grosche , and G. Kerth . 2019. “Differences in Seasonal Survival Suggest Species‐Specific Reactions to Climate Change in Two Sympatric Bat Species.” Ecology and Evolution 9, no. 14: 7957–7965. 31380063 10.1002/ece3.5292 PMC6662409

[gcb70531-bib-0056] Richardson, P. 1994. “A New Method of Distinguishing Daubenton's Bats (*Myotis daubentonii*) up to One Year Old From Adults.” Journal of Zoology 233, no. 2: 307–309.

[gcb70531-bib-0057] Ruf, T. , and C. Bieber . 2020. “Physiological, Behavioral, and Life‐History Adaptations to Environmental Fluctuations in the Edible Dormouse.” Frontiers in Physiology 11: 423. 32431626 10.3389/fphys.2020.00423 PMC7214925

[gcb70531-bib-0058] Russo, D. , and B. Fenton . 2023. A Natural History of Bat Foraging: Evolution, Physiology, Ecology, Behavior, and Conservation. Elsevier.

[gcb70531-bib-0059] Salvarina, I. , D. Gravier , and K.‐O. Rothhaupt . 2017. “Seasonal Insect Emergence From Three Different Temperate Lakes.” Limnologica 62: 47–56.

[gcb70531-bib-0060] Senior, P. , R. K. Butlin , and J. D. Altringham . 2005. “Sex and Segregation in Temperate Bats.” Proceedings of the Royal Society B: Biological Sciences 272, no. 1580: 2467–2473. 10.1098/rspb.2005.3237 PMC1599787 16271970

[gcb70531-bib-0061] Spitzenberger, F. , K. Kugelschafter , and E. Weiss . 2023. “Hibernation Phenologies and Winter Activities of Four Congeneric Bat Species Hibernating Simultaneously in an Eastern Alpine Cave Studied by Phototrapping (Chiroptera: Vespertilionidae).” Lynx, Series Nova 54, no. 1: 155–172.

[gcb70531-bib-0062] Stepanian, P. M. , and C. E. Wainwright . 2018. “Ongoing Changes in Migration Phenology and Winter Residency at Bracken Bat Cave.” Global Change Biology 24, no. 7: 3266–3275. 29442413 10.1111/gcb.14051

[gcb70531-bib-0063] Thackeray, S. J. , P. A. Henrys , D. Hemming , et al. 2016. “Phenological Sensitivity to Climate Across Taxa and Trophic Levels.” Nature 535, no. 7611: 241–245. 27362222 10.1038/nature18608

[gcb70531-bib-0064] Turbill, C. , C. Bieber , and T. Ruf . 2011. “Hibernation Is Associated With Increased Survival and the Evolution of Slow Life Histories Among Mammals.” Proceedings of the Royal Society B: Biological Sciences 278, no. 1723: 3355–3363. 10.1098/rspb.2011.0190 PMC3177628 21450735

[gcb70531-bib-0065] Turbill, C. , and S. Prior . 2016. “Thermal Climate‐Linked Variation in Annual Survival Rate of Hibernating Rodents: Shorter Winter Dormancy and Lower Survival in Warmer Climates.” Functional Ecology 30, no. 8: 1366–1372.

[gcb70531-bib-0066] Vallance, N. , V. Zhang , and C. L. Buck . 2025. “Preparing for Hibernation: Above Ground Activity and Body Temperature of Free‐Living Golden Mantled Ground Squirrels ( *Callospermophilus lateralis* ).” *Canadian Journal of Zoology*. 10.1139/cjz-2024-0149.

[gcb70531-bib-0067] van Schaik, J. , R. Janssen , T. Bosch , A.‐J. Haarsma , J. J. Dekker , and B. Kranstauber . 2015. “Bats Swarm Where They Hibernate: Compositional Similarity Between Autumn Swarming and Winter Hibernation Assemblages at Five Underground Sites.” PLoS One 10, no. 7: e0130850. 26153691 10.1371/journal.pone.0130850 PMC4496085

[gcb70531-bib-0068] Vuarin, P. , and P.‐Y. Henry . 2014. “Field Evidence for a Proximate Role of Food Shortage in the Regulation of Hibernation and Daily Torpor: A Review.” Journal of Comparative Physiology B 184: 683–697. 10.1007/s00360-014-0833-0 24850375

[gcb70531-bib-0069] Wells, C. P. , R. Barbier , S. Nelson , R. Kanaziz , and L. M. Aubry . 2022. “Life History Consequences of Climate Change in Hibernating Mammals: A Review.” Ecography 2022, no. 6: e06056.

[gcb70531-bib-0070] Williams, C. T. , B. M. Barnes , G. J. Kenagy , and C. L. Buck . 2014. “Phenology of Hibernation and Reproduction in Ground Squirrels: Integration of Environmental Cues With Endogenous Programming.” Journal of Zoology 292, no. 2: 112–124.

[gcb70531-bib-0071] Williams, C. T. , K. Wilsterman , V. Zhang , J. Moore , B. M. Barnes , and C. L. Buck . 2016. “The Secret Life of Ground Squirrels: Accelerometry Reveals Sex‐Dependent Plasticity in Above‐Ground Activity.” Royal Society Open Science 3, no. 9: 160404. 27703706 10.1098/rsos.160404 PMC5043325

[gcb70531-bib-0072] Willis, C. K. 2017. “Trade‐Offs Influencing the Physiological Ecology of Hibernation in Temperate‐Zone Bats.” Integrative and Comparative Biology 57, no. 6: 1214–1224. 28985332 10.1093/icb/icx087

