# Supplementary material for: One Species Hibernates Shorter, the Other Longer: Rapid but Opposing Responses to Warming Climate in Two Sympatric Bat Species
Source: Glob Chang Biol. 2025 Oct 2;31(10):e70531. doi: 10.1111/gcb.70531 (PMC12491832; doi:10.1111/gcb.70531)
Supplement: Supplementary file 1 — Tables S1–S3: gcb70531‐sup‐0001‐TableS1‐S3.pdf. [file GCB-31-e70531-s001.pdf]

Supplementary materials to:

Krivek, G. & Meier, F., Grosche, L., Kerth, G. & van Schaik, J. (2025). **One Species Hibernates Shorter, the Other Longer: Rapid but Opposing Responses to Warming Climate in Two Sympatric Bat Species.** *Global Change Biology*.

Table S1. Sample size of all individuals per species, sex-age subgroup, and year used for analysing long-term patterns of hibernation phenology (i.e., start, end, and duration of longest hibernation period) for two sympatric bat species (*Myotis daubentonii* and *M. nattereri*). Years marked with * were excluded from parts of the analysis: year 2016/17 for hibernation end and duration of *M. daubentonii* and *M. nattereri*, 2017/18 and 2018/19 for hibernation start and duration of *M. nattereri*.

Year	*Myotis daubentonii*				*Myotis nattereri*			
	adult females	adult males	juvenile females	juvenile males	adult females	adult males	juvenile females	juvenile males
2010/11	48	17	17	15	38	13	11	15
2011/12	86	72	17	21	78	50	25	42
2012/13	93	103	11	7	115	83	21	29
2013/14	102	120	13	22	124	105	17	42
2014/15	113	159	18	15	165	141	20	20
2015/16	89	141	0	0	138	128	0	0
2016/17*	75	108	7	13	113	121	9	23
2017/18*	33	52	5	13	37	44	8	8
2018/19*	44	78	12	12	30	37	5	11
2019/20	97	152	12	10	96	72	13	15
2020/21	65	123	7	21	91	86	14	21
2021/22	54	90	11	6	40	35	2	3
2022/23	52	77	10	8	39	28	2	3

Table S2. Summary of linear mixed-effects model structures and outputs used to analyse hibernation phenology (start, end and duration) of *Myotis daubentonii* and *M. nattereri*. For each model, fixed effect estimates, standard errors, degrees of freedom (df), t-values, and p-values are reported, along with variance components for random effects. Models were fitted using REML in the *lme4* package in R, and p-values were computed using Satterthwaite's method via the *lmerTest* package.

A) Hibernation start of *Myotis daubentonii*

Formula: **Mdau_hibernation_start_date ~ year + sex_age + yeart:sex_age + (1 | ID)**						
Fixed effects:						
	**Estimate**	**Std Error**	**df**	**t value**	**Pr(>|t|)**	
(Intercept)	**75.5153**	**1.5156**	**1782.701**	**49.827**	**< 2e-16**	***
year	**-2.1424**	**0.171**	**2430.59**	**-12.526**	**< 2e-16**	***
sex_age_adult_female	**-15.2411**	**2.2042**	**1608.047**	**-6.915**	**6.75E-12**	***
sex_age_juvenile_female	2.5633	3.289	2463.874	0.779	0.435842	
sex_age_juvenile_male	**9.4945**	**3.0612**	**2485.783**	**3.102**	**0.001947**	**
year:sex_age_adult_female	**1.1766**	**0.2602**	**2279.278**	**4.523**	**6.42E-06**	***
year:sex_age_juvenile_female	**1.4452**	**0.4338**	**2456.019**	**3.331**	**0.000877**	***
year:sex_age_juvenile_male	**1.4885**	**0.4153**	**2530.446**	**3.584**	**0.000344**	***

Random effects:			
**Groups**	**Name**	**Variance**	**Std Dev**
ID	(Intercept)	160.60	12.67
Residual		241.50	15.54
Number of obs: 2546, groups: ID, 948			

B) Hibernation end of *Myotis daubentonii*

Formula: **Mdau_hibernation_end_date ~ year + sex_age + yeart:sex_age + (1 | ID)**						
Fixed effects:						
	**Estimate**	**Std Error**	**df**	**t value**	**Pr(>|t|)**	
(Intercept)	**224.7188**	**1.3214**	**1478.763**	**170.06**	**< 2e-16**	***
year	-0.1627	0.1519	2079.893	-1.071	0.28422	
sex_age_adult_female	**4.3055**	**1.9114**	**1316.541**	**2.253**	**0.02445**	*
sex_age_juvenile_female	**-5.8261**	**2.93**	**2276.802**	**-1.988**	**0.04688**	*
sex_age_juvenile_male	**-7.8714**	**2.7719**	**2282.743**	**-2.84**	**0.00456**	**
year:sex_age_adult_female	-0.04	0.2299	1895.117	-0.174	0.86189	
year:sex_age_juvenile_female	0.3527	0.385	2269.602	0.916	0.35971	
year:sex_age_juvenile_male	**0.9755**	**0.3711**	**2332.844**	**2.629**	**0.00863**	**

Random effects:			
**Groups**	**Name**	**Variance**	**Std Dev**
ID	(Intercept)	94.79	9.74
Residual		207.05	14.39
Number of obs: 2343, groups: ID, 930			

C) Hibernation duration of *Myotis daubentonii*

Formula: **Mdau_hibernation_duration ~ year + sex_age + yeart:sex_age + (1 | ID)**						
Fixed effects:						
	**Estimate**	**Std Error**	**df**	**t value**	**Pr(>|t|)**	
(Intercept)	**150.0531**	**2.2241**	**1613.617**	**67.467**	**< 2e-16**	***
year	**1.8068**	**0.253**	**2179.538**	**7.142**	**1.25E-12**	***
sex_age_adult_female	**19.0773**	**3.2267**	**1445.297**	**5.912**	**4.20E-09**	***
sex_age_juvenile_female	**-10.0408**	**4.862**	**2285.792**	**-2.065**	**0.03902**	*
sex_age_juvenile_male	**-17.6822**	**4.5636**	**2273.646**	**-3.875**	**0.00011**	***
year:sex_age_adult_female	**-1.1571**	**0.3842**	**2015.735**	**-3.011**	**0.00263**	**
year:sex_age_juvenile_female	-0.9891	0.6391	2276.885	-1.548	0.12185	
year:sex_age_juvenile_male	-0.4811	0.6139	2332.875	-0.784	0.43326	

Random effects:			
**Groups**	**Name**	**Variance**	**Std Dev**
ID	(Intercept)	309.10	17.58
Residual		541.40	23.27
Number of obs: 2343, groups: ID, 930			

D) Hibernation start of *Myotis nattereri*

Formula: **Mnat_hibernation_start_date ~ year + sex_age + yeart:sex_age + (1 | ID)**						
Fixed effects:						
	**Estimate**	**Std Error**	**df**	**t value**	**Pr(>|t|)**	
(Intercept)	**128.6463**	**1.45306**	**1560.094**	**88.535**	**< 2e-16**	***
year	**1.62908**	**0.18708**	**2143.387**	**8.708**	**< 2e-16**	***
sex_age_adult_female	**-10.8295**	**1.93874**	**1461.059**	**-5.586**	**2.77E-08**	***
sex_age_juvenile_female	**-13.1498**	**2.84438**	**2188.649**	**-4.623**	**4.00E-06**	***
sex_age_juvenile_male	**-4.7301**	**2.25484**	**2101.167**	**-2.098**	**0.036**	*
year:sex_age_adult_female	**-1.0844**	**0.25005**	**2101.662**	**-4.337**	**1.51E-05**	***
year:sex_age_juvenile_female	0.09118	0.44402	2187.399	0.205	0.837	
year:sex_age_juvenile_male	-0.54734	0.37007	2236.5	-1.479	0.139	

Random effects:			
**Groups**	**Name**	**Variance**	**Std Dev**
ID	(Intercept)	92.44	9.62
Residual		188.08	13.71
Number of obs: 2246, groups: ID, 857			

E) Hibernation end of *Myotis nattereri*

Formula: **Mnat_hibernation_end_date ~ year + sex_age + yeart:sex_age + (1 | ID)**						
Fixed effects:						
	**Estimate**	**Std Error**	**df**	**t value**	**Pr(>|t|)**	
(Intercept)	**209.3246**	**1.33548**	**1610.725**	**156.741**	**< 2e-16**	***
year	**-0.54499**	**0.17063**	**2072.024**	**-3.194**	**0.00142**	**
sex_age_adult_female	**11.74936**	**1.78531**	**1497.127**	**6.581**	**6.44E-11**	***
sex_age_juvenile_female	0.3008	2.61929	2122.372	0.115	0.90858	
sex_age_juvenile_male	3.54646	2.07405	2006.074	1.71	0.08743	.
year:sex_age_adult_female	-0.40813	0.22854	2026.794	-1.786	0.07428	.
year:sex_age_juvenile_female	0.30153	0.39832	2116.918	0.757	0.44914	
year:sex_age_juvenile_male	-0.07354	0.33002	2147.417	-0.223	0.82369	

Random effects:			
**Groups**	**Name**	**Variance**	**Std Dev**
ID	(Intercept)	76.71	8.76
Residual		161.14	12.69
Number of obs: 2160, groups: ID, 851			

F) Hibernation duration of *Myotis nattereri*

Formula: **Mnat_hibernation_duration ~ year + sex_age + yeart:sex_age + (1 | ID)**						
Fixed effects:						
	**Estimate**	**Std Error**	**df**	**t value**	**Pr(>|t|)**	
(Intercept)	**80.8397**	**1.9481**	**1463.43**	**41.497**	**< 2e-16**	***
year	**-2.3437**	**0.2481**	**1911.829**	**-9.447**	**< 2e-16**	***
sex_age_adult_female	**21.9161**	**2.6113**	**1371.454**	**8.393**	**< 2e-16**	***
sex_age_juvenile_female	**12.7415**	**3.7148**	**1943.77**	**3.43**	**0.000616**	***
sex_age_juvenile_male	**8.0519**	**2.8927**	**1796.191**	**2.783**	**0.005434**	**
year:sex_age_adult_female	**0.8319**	**0.3321**	**1884.712**	**2.505**	**0.012343**	*
year:sex_age_juvenile_female	0.5141	0.5855	1937.349	0.878	0.380044	
year:sex_age_juvenile_male	0.544	0.491	1970.051	1.108	0.268074	

Random effects:			
**Groups**	**Name**	**Variance**	**Std Dev**
ID	(Intercept)	201.70	14.20
Residual		292.50	17.10
Number of obs: 1980, groups: ID, 807			

Table S3. Summary of linear mixed-effects model structures and outputs used to analyse the effect of median air temperature on the hibernation start and end dates of *Myotis daubentonii* and *M. nattereri*. For each model, fixed effect estimates, standard errors, degrees of freedom (df), t-values, and p-values are reported, along with variance components for random effects. Models were fitted using REML in the *lme4* package in R, and p-values were computed using Satterthwaite's method via the *lmerTest* package.

A) Median hibernation start date of *M. daubentonii* and median temperature in August and September

Formula: **Mdau_median_hibernation_start ~ median_aug_sept_temp * sex_age + (1 | year)**						
Fixed effects:						
	**Estimate**	**Std Error**	**df**	**t value**	**Pr(>|t|)**	
(Intercept)	**158.282**	**26.503**	**33.318**	**5.972**	**1.01E-06**	***
median_aug_sept_temp	**-6.689**	**1.612**	**33.318**	**-4.15**	**0.000216**	***
sex_age_adult_male	50.476	31.415	30.905	1.607	0.118287	
sex_age_juvenile_female	-17.851	31.551	30.993	-0.566	0.575613	
sex_age_juvenile_male	-60.461	31.551	30.993	-1.916	0.06459	.
median_aug_sept_temp:sex_age_adult_male	-2.559	1.91	30.905	-1.339	0.190213	
median_aug_sept_temp:sex_age_juvenile_female	2.539	1.917	30.971	1.325	0.195028	
median_aug_sept_temp:sex_age_juvenile_male	**5.616**	**1.917**	**30.971**	**2.93**	**0.006312**	**

Random effects:			
**Groups**	**Name**	**Variance**	**Std Dev**
year	(Intercept)	8.16	2.86
Residual		19.27	4.39
Number of obs: 50, groups: year, 13			

B) Median hibernation end date of *M. daubentonii* and median temperature in February and March

Formula: **Mdau_ median_hibernation_end ~ median_feb_mar_temp * sex_age + (1 | year)**						
Fixed effects:						
	**Estimate**	**Std Error**	**df**	**t value**	**Pr(>|t|)**	
(Intercept)	**234.8069**	**4.5746**	**32.2444**	**51.328**	**<2e-16**	***
median_feb_mar_temp	-0.2117	0.8592	32.2444	-0.246	0.8069	
sex_age_adult_male	**-12.0646**	**5.5817**	**28.4618**	**-2.161**	**0.0392**	*
sex_age_juvenile_female	-9.921	5.6362	28.6482	-1.76	0.089	.
sex_age_juvenile_male	-10.7974	5.6362	28.6482	-1.916	0.0654	.
median_aug_sept_temp:sex_age _adult_male	0.6139	1.0483	28.4618	0.586	0.5627	
median_aug_sept_temp:sex_age _juvenile_female	0.1717	1.0503	28.4992	0.163	0.8713	
median_aug_sept_temp:sex_age _juvenile_male	0.3638	1.0503	28.4992	0.346	0.7316	

Random effects:			
**Groups**	**Name**	**Variance**	**Std Dev**
year	(Intercept)	7.78	2.79
Residual		22.64	4.76
Number of obs: 46, groups: year, 12			

C) Median hibernation start date of *M. nattereri* and median temperature in October and November

Formula: **Mnat_median_hibernation_start ~ median_oct_nov_temp * sex_age + (1 | year)**						
Fixed effects:						
	**Estimate**	**Std Error**	**df**	**t value**	**Pr(>|t|)**	
(Intercept)	**107.873**	**19.186**	**27.914**	**5.623**	**5.13E-06**	***
median_oct_nov_temp	1.352	2.123	27.914	0.637	0.5293	
sex_age_adult_male	-23.986	23.004	25.315	-1.043	0.307	
sex_age_juvenile_female	-34.231	23.05	25.348	-1.485	0.1499	
sex_age_juvenile_male	-20.812	23.05	25.348	-0.903	0.3751	
median_oct_nov_temp:sex_age _adult_male	4.793	2.545	25.315	1.883	0.0712	.
median_oct_nov_temp:sex_age _juvenile_female	4.354	2.556	25.39	1.703	0.1007	
median_oct_nov_temp:sex_age _juvenile_male	3.433	2.556	25.39	1.343	0.1912	

Random effects:			
**Groups**	**Name**	**Variance**	**Std Dev**
year	(Intercept)	17.8	4.22
Residual		45.5	6.75
Number of obs: 42, groups: year, 11			

D) Median hibernation end date of *M. nattereri* and median temperature in January and February

<table>
<tr><td colspan="7">Formula: Mnat_ median_hibernation_end ~ median_jan_feb_temp * sex_age + (1 | year)</td></tr>
<tr><td>Fixed effects:</td><td></td><td></td><td></td><td></td><td></td><td></td></tr>
<tr><td></td><td>Estimate</td><td>Std Error</td><td>df</td><td>t value</td><td>Pr(>|t|)</td><td></td></tr>
<tr><td>(Intercept)</td><td>218.8815</td><td>6.58716</td><td>34.93615</td><td>33.229</td><td><2e-16</td><td>***</td></tr>
<tr><td>median_jan_feb_temp</td><td>-1.2251</td><td>1.6381</td><td>34.93615</td><td>-0.748</td><td>0.46</td><td></td></tr>
<tr><td>sex_age_adult_male</td><td>-8.61338</td><td>8.44184</td><td>28.48143</td><td>-1.02</td><td>0.316</td><td></td></tr>
<tr><td>sex_age_juvenile_female</td><td>-6.11617</td><td>8.47568</td><td>28.56967</td><td>-0.722</td><td>0.476</td><td></td></tr>
<tr><td>sex_age_juvenile_male</td><td>-4.80578</td><td>8.47568</td><td>28.56967</td><td>-0.567</td><td>0.575</td><td></td></tr>
<tr><td>median_oct_nov_temp:sex_age_adult_male</td><td>0.03054</td><td>2.09932</td><td>28.48143</td><td>0.015</td><td>0.988</td><td></td></tr>
<tr><td>median_oct_nov_temp:sex_age_juvenile_female</td><td>-0.30583</td><td>2.09933</td><td>28.48147</td><td>-0.146</td><td>0.885</td><td></td></tr>
<tr><td>median_oct_nov_temp:sex_age_juvenile_male</td><td>0.90802</td><td>2.09933</td><td>28.48147</td><td>0.433</td><td>0.669</td><td></td></tr>
</table>

Random effects:			
**Groups**	**Name**	**Variance**	**Std Dev**
year	(Intercept)	13.75	3.71
Residual		63.13	7.95
Number of obs: 46, groups: year, 12			
